# Functional MRI reveals regional changes of brain activity in rats following longitudinal focal high-density theta burst stimulation (hdTBS)

**DOI:** 10.1162/IMAG.a.92

**Published:** 2025-07-25

**Authors:** Charlotte Qiong Li, Samantha Hoffman, Hieu Nguyen, Antonia Vrana, Aidan Carney, Ying Duan, Zilu Ma, Nanyin Zhang, Yihong Yang, Hanbing Lu

**Affiliations:** Department of Biomedical Engineering, The Pennsylvania State University, University Park, PA, United States; Neuroimaging Research Branch, National Institute on Drug Abuse (NIDA) Intramural Research Program, Baltimore, MD, United States; Center for Neurotechnology in Mental Health Research, The Pennsylvania State University, University Park, PA, United States; Center for Neural Engineering, The Pennsylvania State University, University Park, PA, United States

**Keywords:** MEP, CBV, motor cortex, TMS, metabolism, fMRI

## Abstract

The therapeutic benefits of transcranial magnetic stimulation (TMS) are believed to stem from neuroplasticity induced by repeated sessions. While animal models have contributed to our understanding of TMS-induced plasticity, there is a need for a rodent model that closely replicates the prolonged conditions experienced by humans. This study aimed to develop a rat model that reflects the spatial and temporal dynamics of human TMS protocols and to evaluate the carryover effects of TMS on the brain at a systems level. Experiments were carried out on two groups of rats (N = 33). In the first cohort, rats were implanted with microwire electrodes to record motor-evoked potential (MEP) signals and received daily sessions of high-density theta burst stimulation (hdTBS) for 5 days. Cortical excitability was assessed through input-output (I-O) curves before and after hdTBS (Day 0 and Day 6). To identify brain regions affected by the longitudinal TMS, the second cohort underwent identical TMS protocol and received fMRI scans on Days 0 and 6 to measure basal cerebral blood volume (CBV). Results reveal that daily hdTBS significantly shifted I-O curves upward in the TMS group (N = 9) compared to the sham group (N = 7), reflecting enhanced cortical excitability. Additionally, fMRI data showed elevated basal CBV in both the stimulation sites and in the connected networks (N = 8 for active TMS and N = 9 for sham), suggesting increased basal metabolism. This study opens a novel platform for further exploring the mechanisms underlying TMS-induced plasticity.

## 1 Introduction

Therapeutic efficacy and time efficiency are two important factors in clinical application of transcranial magnetic stimulation (TMS). High-frequency repetitive TMS (rTMS) at 10 Hz and intermittent theta burst stimulation (iTBS) are the two most widely used protocols clinically. iTBS administers 600 pulses over 190 seconds, as opposed to 3000 pulses over 37.5 minutes in 10 Hz rTMS (Huang et al., 2005). Both paradigms achieve similar, although modest, efficacy in treatment-resistant major depression (Berlim et al., 2014; Blumberger et al., 2018; Downar et al., 2016; Eshel et al., 2020). The Stanford Neuromodulation Therapy (SNT) paradigm (Cole et al., 2022), an “accelerated” version of iTBS, achieved a higher remission rate. SNT applies 10 TMS sessions per day, and each session delivers 1800 iTBS pulses with an inter-session interval of 50 minutes. Note that conventional iTBS was the building block of the SNT paradigm, which has been shown to exhibit limited effect in modulating cortical excitability (Tiksnadi et al., 2020). There is a need to develop new TBS technology to enhance the efficacy while maintaining high time-efficiency, and to support further treatment optimization. For example, replacing the iTBS sessions in SNT with more effective TBS paradigms could potentially reduce the number of sessions required in a day while achieving high efficacy.

The therapeutic effects of TMS are believed to result from neuroplasticity that occurs over multiple sessions of TMS administration. For ethical reasons, testing of a new paradigm in healthy participants is usually limited to evaluating the after-effects in the motor cortex (Huang et al., 2005; Matsumoto & Ugawa, 2020). Therapeutic potential of the new paradigm is then assessed in patients, largely on a trial-and-error basis. A more systematic approach might involve developing TMS technology and assessing its acute and carryover effects in animal models first, with the aim of translating those results to human applications.

The translation of TMS protocols from humans to animals presents several technical challenges, with coil focality being a primary concern (Wilson et al., 2018). Small laboratory animals such as rats and mice require a small TMS coil to minimize off-target effects (Deng et al., 2013); however, a small coil has a low efficiency (Cohen et al., 1990; Deng et al., 2013), necessitating exceedingly high electric current to induce a suprathreshold response in the brain. This leads to additional challenges such as coil overheating and electromagnetic stress. Recently, we developed a rodent-specific TMS coil with a focality of 2 mm (Cermak et al., 2020; Meng et al., 2018); Additionally, we introduced a high-density TBS (hdTBS) paradigm that administers up to 6 pulses per burst, as opposed to the 3 pulses in conventional TBS (Meng et al., 2022). We assessed the acute after-effects of hdTBS based on motor evoked potential (MEP) readouts from awake rats, finding that 6-pulse hdTBS enhanced the after-effects by 92% compared to conventional 3-pulse iTBS.

Since longitudinal TMS, rather than acute TMS, holds greater therapeutic potential, in the present study, we aimed to determine whether longitudinal hdTBS sessions can change basal cortical excitability in healthy adult rats. Our previous observations indicated that an acute hdTBS session induced after-effects in healthy adult rats that last for about 35 minutes before returning to the pre-TMS baseline, suggesting that the brain circuits were temporally tipped “off-balanced”, and returned to balanced operation afterwards. Assuming that neural circuits in healthy brains operate at optimal efficiency as a result of evolution, and thus might be resistant to neuromodulation, the demonstration of neuroplasticity in healthy rats through longitudinal TMS would not only establish a model to investigate the mechanisms of TMS action, but also creates a novel and potentially significant platform for developing more effective TMS paradigms for human applications.

To this end, leveraging advancements in focal TMS technology (Meng et al., 2018) and awake rat TMS model (Cermak et al., 2020), we administered daily single-session hdTBS to the motor cortex of awake rats for 5 days. We assessed MEP readouts before and after hdTBS modulation and mapped basal cerebral blood volume (CBV) using magnetic resonance imaging (MRI). CBV mapping, based on an exogenous MRI contrast agent, offers high resolution, high sensitivity, and is quantitative. Basal CBV is strongly coupled to basal cerebral blood flow (CBF) (Grubb et al., 1974) and cerebral metabolism (Raichle, 2009), reflecting brain activity. Our data reveal that one hdTBS session per day for 5 days significantly enhanced the excitability of the rat motor cortex, as measured by the input-output (IO) curve; CBV mapping showed that several brain regions, both proximal and distal to the stimulation sites, exhibited heightened basal CBV, suggesting increased regional brain activity. To the best of our knowledge, this is the first study demonstrating basal brain metabolism changes induced by longitudinal, focal TMS of the motor cortex in awake rats. Our study opens a novel avenue for further investigating TMS mechanisms.

## 2 Materials and Methods

### 2.1 Animal preparation

Data were collected from 33 adult male Sprague–Dawley rats (14 to 16 weeks old) obtained from Charles River Labs. Of these, 16 rats were used for the MEP study (n = 9 for hdTBS and n = 7 for sham stimulation) and 17 for CBV mapping (n = 8 for hdTBS and n = 9 for sham stimulation). Surgical preparations are described below. Following surgery, the rats were individually housed under a reversed 12:12 hour light/dark cycle, with ad libitum access to standard rat chow and water. All experimental procedures were conducted following the guidelines established by the Animal Care and Use Committee of the NIDA-IRP and complied with the regulations outlined in the Guide for the Care and Use of Laboratory Animals.

### 2.2 Microelectrode implantation for longitudinal MEP recording

**Fig. 1.**
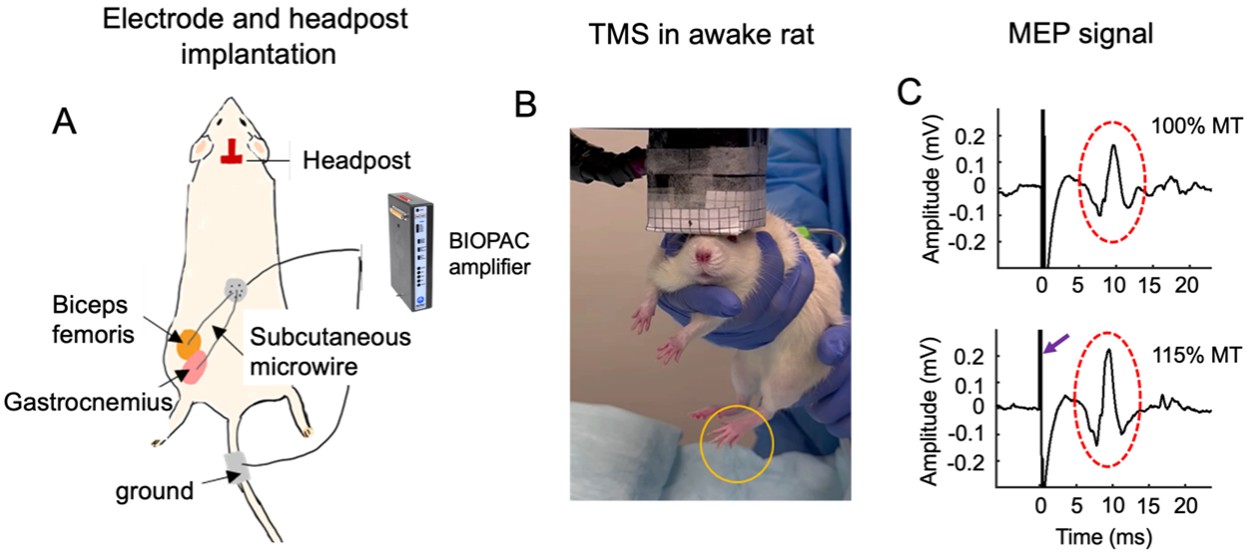
Illustration of experimental setup. (A) Customized microwire electrodes were surgically implanted into the biceps femoris and gastrocnemius muscles of the left hindlimb for longitudinal MEP recording; a headpost was implanted on the rat skull, serving as the reference for consistent coil positioning across TMS sessions and across animals. (B) An awake rat undergoing TMS administration. TMS pulse innervated the left limb (orange circle). (C) shows raw MEP traces at two power levels. The arrow indicates artifacts induced by a TMS pulse; dashed oval circles indicate MEP signal.

MEP has been traditionally employed as a measure to assess the effects of TMS on the motor cortex (Chen et al., 1997; Pascual-Leone et al., 1994). In humans, the MEP signal can be conveniently recorded by measuring the electromyographic (EMG) signal from innervated muscles with surface electrodes. However, this method cannot be readily adapted to awake rats due to strong motion artifacts. We have developed a method that permits consistent MEP recording, detailed previously (Meng et al., 2022), and are illustrated in Figure 1A. Briefly, we customized EMG microwire-electrodes in house. The electrodes were made of soft 7-strand stainless steel microwires (0.025 mm in diameter, A-M systems, Washington, USA, cat. No. 793200), cut into13 cm in length. The insulation coating on one end of the microwires was removed (about 3 mm in length) before being press-connected to a female socket (Model E363/0, P1 Technology, USA). The sockets were then organized and embedded into a 6-channel electrode pedestal (Model: MS363, P1 Technology, USA). To ensure the stability of the sockets and microwires, the pedestal was attached to a circular Marlex mesh secured with dental cement. We custom-made a steel dust cap to cover the pedestal, which helped prevented the animals from chewing the plastic connectors, elongating the lifetime of the instrument during repeated TMS recording sessions. The insulation coating at the other end of microwires was also carefully removed, which were implanted into the biceps femoris muscle and the gastrocnemius muscle of the left hindlimb. These exposed segments served as active electrodes to detect EMG signal in the implanted muscles; one surface EEG pad was attached to rat tail serving as the ground electrode. The two active electrodes and the ground electrode were interfaced to a BIOPAC EMG recording system (BIOPAC Systems Inc, CA, USA). The EMG signal was amplified by a factor of 2000, band-pass filtered between 100 and 5000 Hz, and sampled at 10,000 Hz.

### 2.3 Headpost implantation for consistent coil placement

A homemade TMS coil with a focality of 2 mm was used in this study. The high focality necessitates a method to consistently direct the hotspot of the TMS coil to the targeted brain region (hindlimb motor cortex). We implanted a headpost on the rat skull, which served as the reference to facilitate accurate coil positioning (see Fig. 1A). Detailed procedures for headpost implantation were described previously (Cermak et al., 2020). Briefly, the primary motor cortex of the rat hindlimb has been mapped using classical electrical micro-stimulation (bregma coordinates: -1.8 mm anterior-posteriorly, 2 mm medio-laterally) (Seong et al., 2014). A 3D-printed, T-shaped headpost was carefully implanted onto the rat skull such that the end-pad of the headpost directed the coil hotspot to the rat hindlimb motor cortex. A detachable coil guide of variable thickness can be used to further adjust coil positioning when necessary. The coil center could then be readily aligned to the hindlimb motor cortex by slightly moving the rat’s head along the medio-lateral direction until the left leg twitch was elicited.

### 2.4 Habituation

Following surgery, the rats were allowed to recover before undergoing a 6-day habituation period. Habituation steps were performed to reduce stress during TMS administration. This involved holding the rats under the coil and playing recorded hdTBS sounds three times a day. Following this procedure, the rats showed significantly reduced stress, characterized by the absence of acute stress signs such as screaming, urination, defecation, and attempts to escape.

### 2.5 Longitudinal hdTBS and I-O curve measurement

Following 6 days of habituation to the TMS environment and behavioral handling, we mapped the motor threshold and I-O curves on experimental Day 0. The rats were manually held with their hindlimb motor cortex region positioned under the hotspot of the coil (see Fig. 1B). Their ears were manually blocked to minimize the effects of the acoustic noises from the TMS coil, which prevented the startle responses that would otherwise often be triggered if their ears were not blocked.

Motor threshold (MT), defined as the minimal TMS power to induce motor response in at least 50% of the trials, was determined by observing unilateral hindlimb motor twitches and MEP signal. I-O curves were obtained by delivering single pulses at 75% to 115% of the MT in 5% increment. A total of 8 pulses were delivered at each power level with an inter-pulse interval of 7 seconds. The resulting MEP signal was recorded; a separate channel recorded the trigger signal that registered the timing of the TMS pulses.

**Fig. 2.**
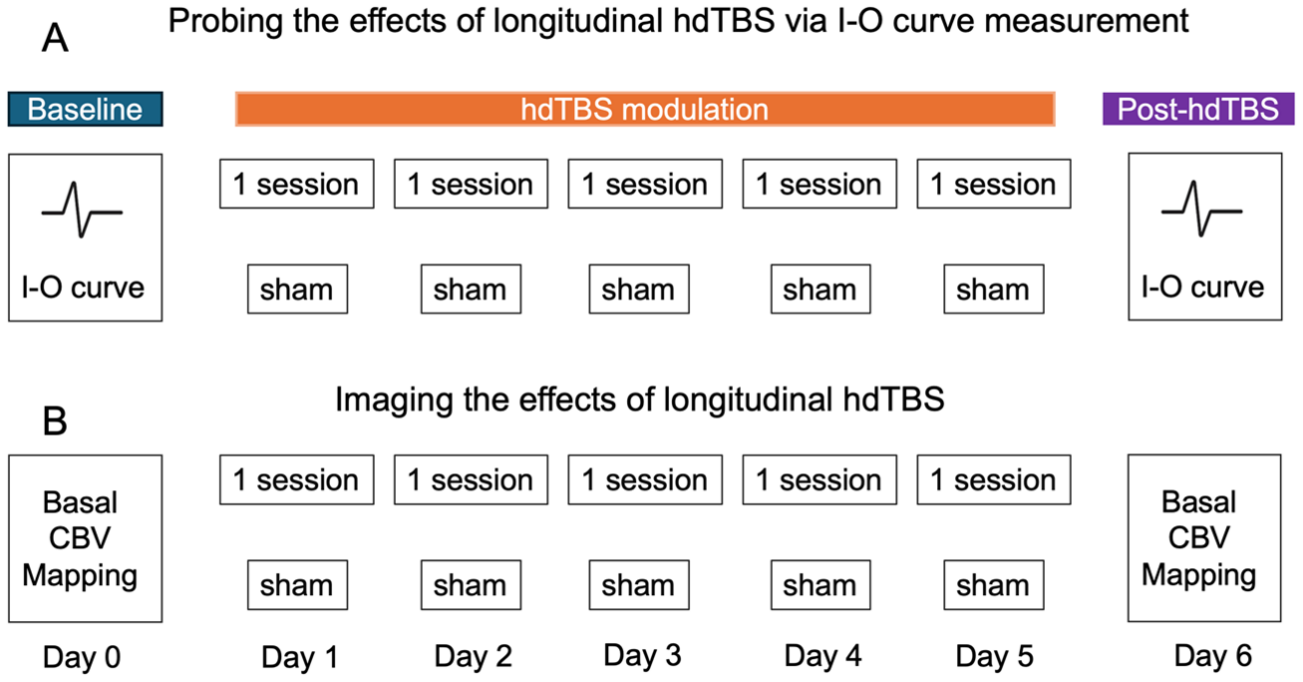
Illustration of experimental design on two separate cohorts of rats. (A) One cohort of rats received daily single-session of either active (N = 8) or sham (N = 7) hdTBS from day 1 to day 5. Input-output (I-O) curves were mapped on Day 0 and Day 6 to assess cortical excitability. (B) Another cohort of rats underwent identical hdTBS modulation procedures as in (A) (N = 8 for active hdTBS; N = 9 for sham hdTBS), fMRI based on CBV mapping was performed on Day 0 and Day 6.

Nine rats received hdTBS sessions from day 1 to day 5. hdTBS was identical to conventional iTBS except that each burst consisted of 6 pulses instead of 3 pulses, delivering a total of 1200 pulses in 190 seconds. TMS power level was set to 100% MT. A separate group of rats (N = 7) received sham TMS. The experimental conditions in the sham group, including electrode/headpost implantation and habituation training, were identical to the TMS group except that the TMS power was set at 70% MT and the rat head was placed 4 cm beneath the TMS coil; the resulting electric field inside the rat brain was practically zero. After 5 days of hdTBS administration, I-O curves were mapped on day 6 following identical procedures as described above. Figure 2A illustrates experimental timeline.

### 2.6 Longitudinal hdTBS and MRI experiments

After determining that 1 hdTBS session/day for 5 days significantly enhanced cortical excitability as probed by I-O curves (see Section 3), we performed MRI experiments on another cohort of rats (N = 8 for TMS, N = 9 for sham). These rats underwent hdTBS or sham treatment using procedures as described above and received fMRI scans on days 0 and 6 under anesthesia (Fig. 2B). Because the MEP electrodes were not compatible with MRI, no electrodes were implanted on these animals; as a result, no I-O curve mapping was performed on these animals.

### 2.7 MRI scan

A combination of isoflurane and dexmedetomidine was used to anesthetize rats during fMRI experiments; detailed procedures were described in (Brynildsen et al., 2016; Lu et al., 2012). MRI data were acquired with a Bruker Biospin 9.4T scanner running the Paravision 7.0 software platform (Bruker Medizintechnik, Karlsruhe, Germany). A volume quadrature transmitter coil (model: MT0381) was used for RF excitation, and a circular surface coil (model: MT0105-20) for MR signal reception. The decussation of the anterior commissure (approximately −0.36 mm from bregma) served as the fiducial landmark to standardize slice localization both within and across animals (Lu, Xi, et al., 2007).

Structural images were collected using a Rapid Acquisition with Relaxation Enhancement sequence. Scan parameters: repetition time (TR) = 3100 ms, echo time (TE) = 36 ms, field of view (FOV) = 30 × 30 cm^2^, in-plane matrix size = 256 × 256, slice thickness = 0.6 mm, slice gap = 0.1 mm, slice number = 31.

Resting-state fMRI data were collected using gradient-echo echo-planner imaging (EPI) sequence developed in-house. fMRI data will be reported elsewhere. CBV-fMRI images were acquired after the resting scans, and they were collected using conventional multi-echo gradient-echo imaging (MGE) sequence. Scan parameters were: TR = 600 ms, TE = 2 ms, 10 echo images, 7 repetitions, slice number = 19, FOV = 30 × 30 mm^2^, and image size = 128 × 128. Immediately after the first two repetitions of data had been acquired, Monocrystalline Iron Oxide Nanoparticles (MION) agent was injected into the tail vein in 1 minute through an infusion pump (Feraheme, AMAG Pharmaceuticals Inc. Cambridge, MA, iron dose = 15 mg/kg). The MION dose was chosen based on literature data (Moreno et al., 2006; Pohlmann et al., 2014) and our experiments (Lu, Scholl, et al., 2007), which was found to provide a good compromise between sensitivity and CBV weighting. MRI data acquisition was uninterrupted while MION infusion was taking place. The MGE scan lasted for 8 minutes and 58 seconds.

### 2.8 Data analysis

#### 2.8.1 MEP data analysis

I-O curve mapping was conducted at 9 TMS power levels, ranging from 75% to 115% MT, with a step size of 5%. The MEP signal in rats features a latency of approximately 6 ms (Meng et al., 2022; Rotenberg et al., 2010); the time stamps of the trigger signal were used to facilitate MEP identification. The peak-to-peak amplitudes of the MEP signal were quantified and averaged across trials for each power level. The average MEP of each rat at each power level was recorded as the output. The maximum amplitude of the baseline I-O curve was recorded, and the MEPs collected at each power level on different days were normalized based on the maximum baseline value.

The amplitude of MEP signal is known to be non-normally distributed (Goetz et al., 2016; Wassermann, 2002), and we performed non-parametric statistical analyses to assess the effects of neuromodulation by TMS. Specifically, to assess whether there was TMS power-dependence in MEP signal and whether there was a difference between Day 6 and Day 0, two-factorial, Aligned Rank Transform Analysis of Variance (ANOVA), a non-parametric statistical method (Leys & Schumann, 2010), was employed. For both the hdTBS and Sham groups, the TMS POWER levels and the DAY (Day 6 vs. Day 0) were considered as the two factors, followed by post hoc Wilcoxon signed-rank Test between power levels (baseline vs. post-stimulation) at each power level, corrected for multiple comparisons using the Bonferroni method.

To compare the effects of active versus sham hdTBS on I-O curve measurement, for both the active and sham hdTBS groups, we computed the I-O curve differences by subtracting the MEP at Day 0 (baseline) from the MEP at Day 6 (post-treatment), this was performed for each subject. Aligned Rank Transform ANOVA was employed with TREATMENT group (active and sham hdTBS) and TMS POWER levels as the two factors. Additionally, the Area Under the Curve (AUC) of the I-O curves was calculated for both groups. The resulting differences in AUC (ΔAUC) reflect changes in cortical excitability following 5 days of active or sham hdTBS. These ΔAUC values were subject to non-parametric, Mann-Whitney U Test, and p < 0.05 was considered statistically significant.

#### 2.8.2 CBV data analysis

fMRI data analyses were performed using two software packages: Analysis of Neuroimages (AFNI) (R. Cox & Hyde, 1997) and FSL (Smith et al., 2004), along with custom scripts written in MATLAB. Initially, the CBV data underwent alignment with a template as follows: high-resolution RARE anatomical images were skull-stripped, linearly aligned to a template; the resulting transformation matrix was applied to the MGE images. Subsequently, relaxation time *R*2* was derived via a least-squares fitting of the MGE data, employing the following equation (Lu et al., 2004):

$$\log(S)=\log(S_{0})-TE\times R2^{*},$$ [Eq. 1]

where *S* denotes MRI signal intensity, *S*_0_ signifies signal intensity at *TE* = 0, and *TE* represents echo time.

This fitting was performed on a voxel-wise basis. To enhance the signal-to-noise ratio (SNR), the R2* values from the first two repetitions acquired before contrast agent injection (pre-MION) were averaged. Similarly, the R2* values from the last two repetitions (post-MION), obtained after contrast agent injection, were also averaged. Subsequently, the Δ*R*2* was computed by subtracting the pre-MION R2* from the post-MION R2*. Voxel-wise Δ*R*2* values are linearly correlated to corresponding CBV (Boxerman et al., 1995; Mandeville et al., 1998, 2004; Wu et al., 2003).

$$\Delta R2^{*}=k\times [CA]\times CBV$$ [Eq. 2]

where *k* is a constant, and CA is the concentration of the contrast agent in blood.

CBV values at baseline (Day 0) and post-TMS (Day 6) in both the hdTBS treatment group and the sham group were computed in the same fashion. Changes in CBV values resulting from hdTBS or sham treatment were derived by calculating the differences in ΔR2* values between Day 0 and Day 6, which were subject to two-tailed t statistics, followed by corrections for multiple comparisons. This was done as follows: first, 3dFWHMx function was applied to calculate the spatial autocorrelation function (ACF) of the MGE data as a function of radius, and fitted that to a model of the form:

$$ACF\left(r\right)=a*e^{-\;\frac{r^{2}}{2b^{2}}}+\left(1-a\right)*e^{-\;\frac{r}{c}}$$ [Eq. 3]

The outputs of the ACF function parameters *a*, *b*, *c* were then fed into 3dClusSim function to derive the cluster size for a given threshold (p < 0.05, voxels in clusters>38). The above calculations were performed using the AFNI software package (R. Cox, 1996). Note that Equation [3] does not assume a Gaussian distribution in spatial autocorrelation of MRI noise, leading to more robust estimate of the cluster size (R. W. Cox et al., 2017). Regions of interest (ROIs) within the activated clusters were mapped into anatomical regions based on the rat brain atlas (Paxinos & Watson, 2007). Voxel-wise rCBV values within individual ROIs were averaged. rCBV values between groups (active vs. sham TMS), and between days (Day 0 and Day 6) were plotted for visual comparison. For each ROI, we pooled data across all subjects and assessed normality using the Shapiro–Wilk test (p-values: 0.10–0.38). The results indicated that the data were normally distributed, supporting the use of parametric comparisons.

## 3 Results

hdTBS sessions were well tolerated by the animals. We observed no signs of abnormality in their daily behaviors, including eating, drinking or grooming, etc. Importantly, no signs of seizure events were observed in any of the animals during or after hdTBS administration.

As an example, Figure 1C shows raw MEP signals acquired on Day 0 from one representative rat. There were large amplitude artifacts immediately following the TMS pulses lasting for about 4 ms (indicated by the arrow), followed by multi-phasic MEP signal that featured a latency of about 6 ms (indicated by the oval circles).

### 3.1 I-O curves at baseline and 1 day post-hdTBS modulation

**Fig. 3.**
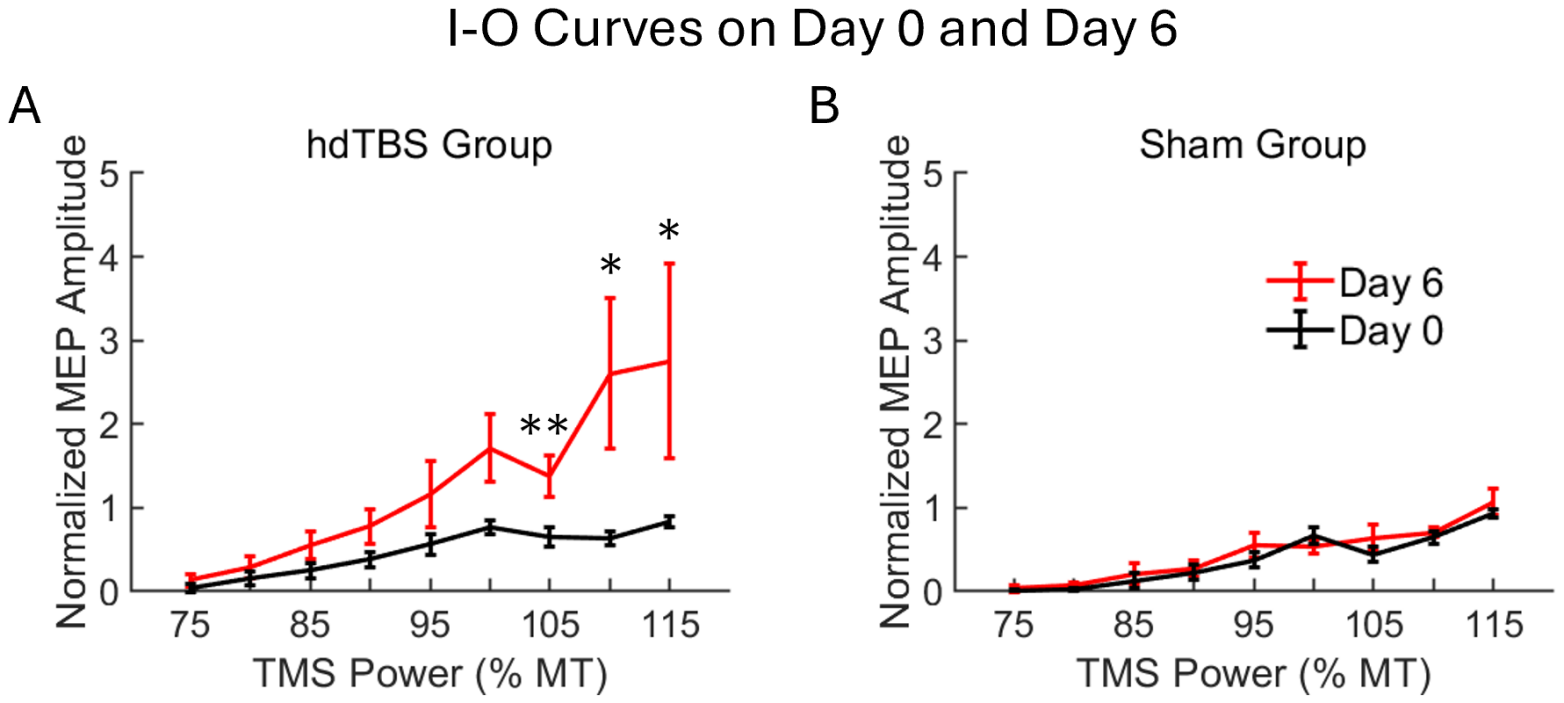
Input-output (I-O) curves were mapped at baseline (Day 0) and 1-day post-hdTBS modulation (Day 6). (A) Daily single-session of hdTBS for 5 days significantly shifted I-O curve upwards, with significant increases observed at stimulation intensities of 105% MT (**p = 0.0029), 110% MT (*p = 0.013), and 115% MT (*p = 0.021). (B) No significant changes in the I-O curve were observed in the sham TMS group.

Figure 3 summarizes I-O curve measurements on Day 0 (baseline) and Day 6 (1 day post-TMS modulation). In the hdTBS group, there was a significant main effect of POWER levels (F = 17.15, p < 2.22 × 10^-16^); there was a significant main effect of DAY (Day 0 and 6) (F = 73.98, p = 1.69 × 10^-14^). Furthermore, there was a significant interaction between POWER and DAY (F = 4.55, p = 6.34 × 10^-5^). Post-hoc Wilcoxon signed-rank tests revealed that significant differences were observed at power levels of 105% MT (p = 0.0029), 110% MT (p = 0.013), and 115% MT (p = 0.021). These findings suggest that MEP responses were significantly altered following hdTBS at these specific power levels, while other levels did not show significant changes. These data reveal that MEP responses were TMS power-level dependent and were significantly modulated by 5 daily sessions of hdTBS administration.

In the sham group, there was a significant main effect of POWER levels (F = 29.84, p < 2 × 10^-16^); but there was no significant main effect of DAY (F = 2.87, p = 0.093), and no significant interaction between POWER levels and DAY (F = 0.71, p = 0.68). These data reveal that MEP responses were significantly modulated by TMS power-levels, but were not significantly modulated by 5 daily sessions of sham hdTBS.

**Fig. 4.**
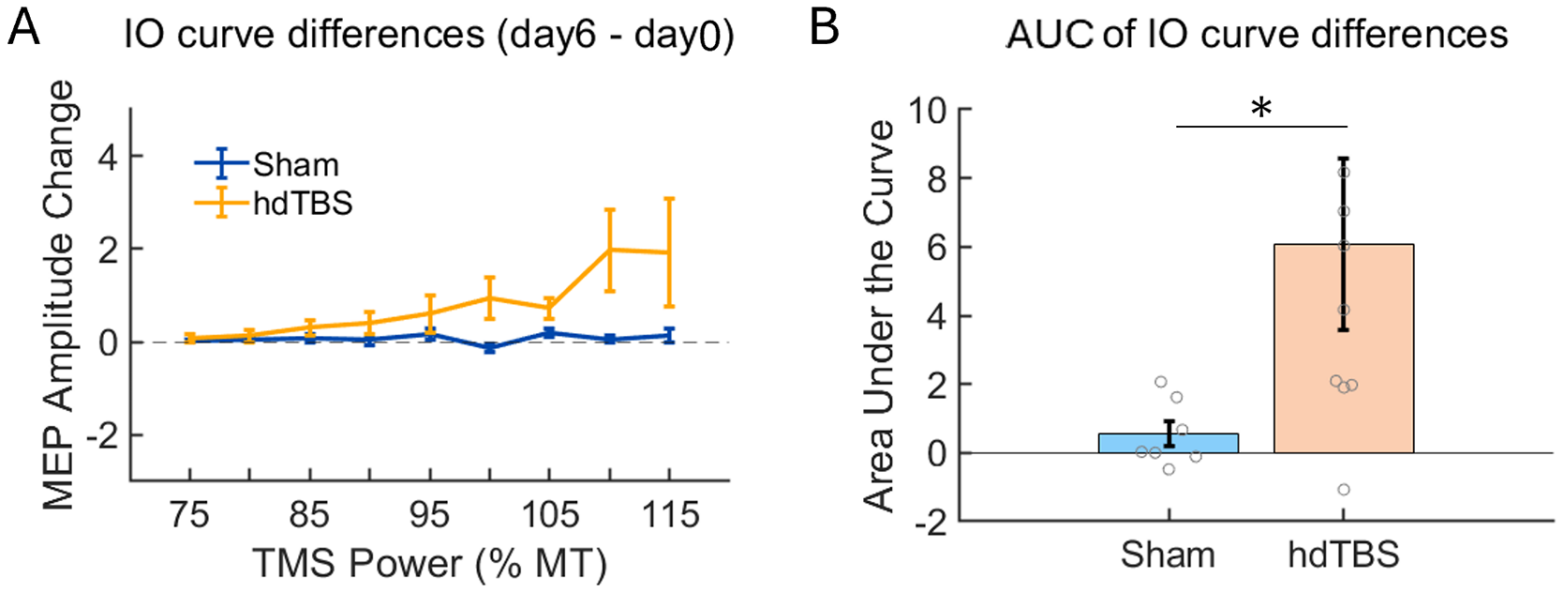
(A) I-O curve differences (delta-IO), calculated by subtracting I-O curves on Day 0 from that on Day 6, in the hdTBS (N = 8) and sham group (N = 7). (B) Area under the curve (AUC) of the delta-IO values in the active and sham hdTBS group (*p = 0.016).

Individual animals’ I-O curves for the hdTBS and sham groups are presented in Figures S1 and S2. To compare the alterations in the I-O curves resulting from active or sham hdTBS, we calculated the difference between the pre-stimulation baseline (Day 0) and post-stimulation (Day 6) curves, yielding delta-IO curves for each animal. These delta-IO curves were subject to two-factorial Aligned Rank Transform ANOVA (TMS POWER × TREATMENT (active/sham hdTBS)). Results are summarized in Figure 4A. There was significant main effect of TREATMENT (F = 31.15, p = 1.44 × 10^-7^); there was significant main effect of POWER (F = 3.943, p = 0.0004); and there was significant POWER × TREATMENT interaction (F = 4.05, p = 0.0003). Furthermore, there was significant difference in AUC values of the delta-IO curves between the active and sham hdTBS groups (Mann-Whitney U test: U = 54, p = 0.016) (Fig. 4B).

### 3.2 CBV mapping

**Fig. 5.**
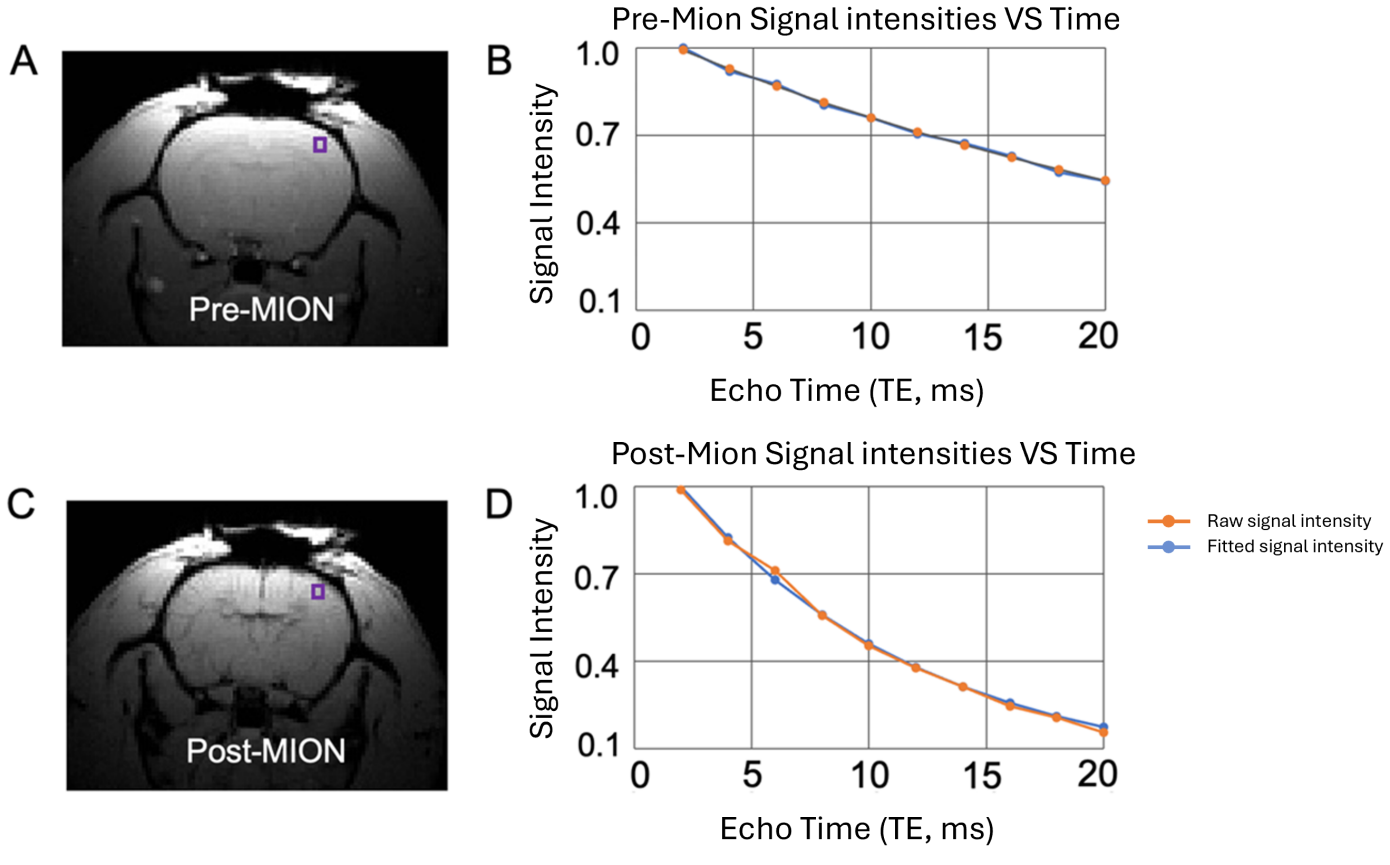
Visual comparison of MRI signal before and after contrast agent injection (MION, iron dose: 15 mg/kg). (A) Representative gradient echo image before MION injection, along with raw (orange) and fitted (blue) MRI signal over various echo times (TEs) shown in (B). (C-D) The same imaging slice after MION injection. MRI signal was chosen from the voxel indicated by the square boxes in (A) and (C), respectively.

Having discovered that daily single-hdTBS session for 5 days significantly shifted the I-O curves upward, indicating enhanced cortical excitability in the stimulated loci, we next investigated whether the functions of the brain regions interconnected with the stimulation loci were also altered by longitudinal TMS. To this end, we mapped basal CBV of the entire rat brain with the injection of intravascular contrast agent MION. As an example, Figure 5A shows a pre-MION raw MGE image (TE = 2 ms); 5B plots voxel-wise MRI signals across TEs (2–20 ms). Figure 5C–D show the same slice post-MION and corresponding signal vs. TE plot. The TE-dependence of the MRI signal can be well described using a single exponential decay model (Fig. 5B, 5D). As shown in this figure, and consistent with previous reports in rats and mice (Lu et al., 2004; Mandeville et al., 1998; Moreno et al., 2006; Pohlmann et al., 2014; Wu et al., 2003), MION injection drastically increased the relaxation rate (R2*) of the MRI signal.

**Fig. 6.**
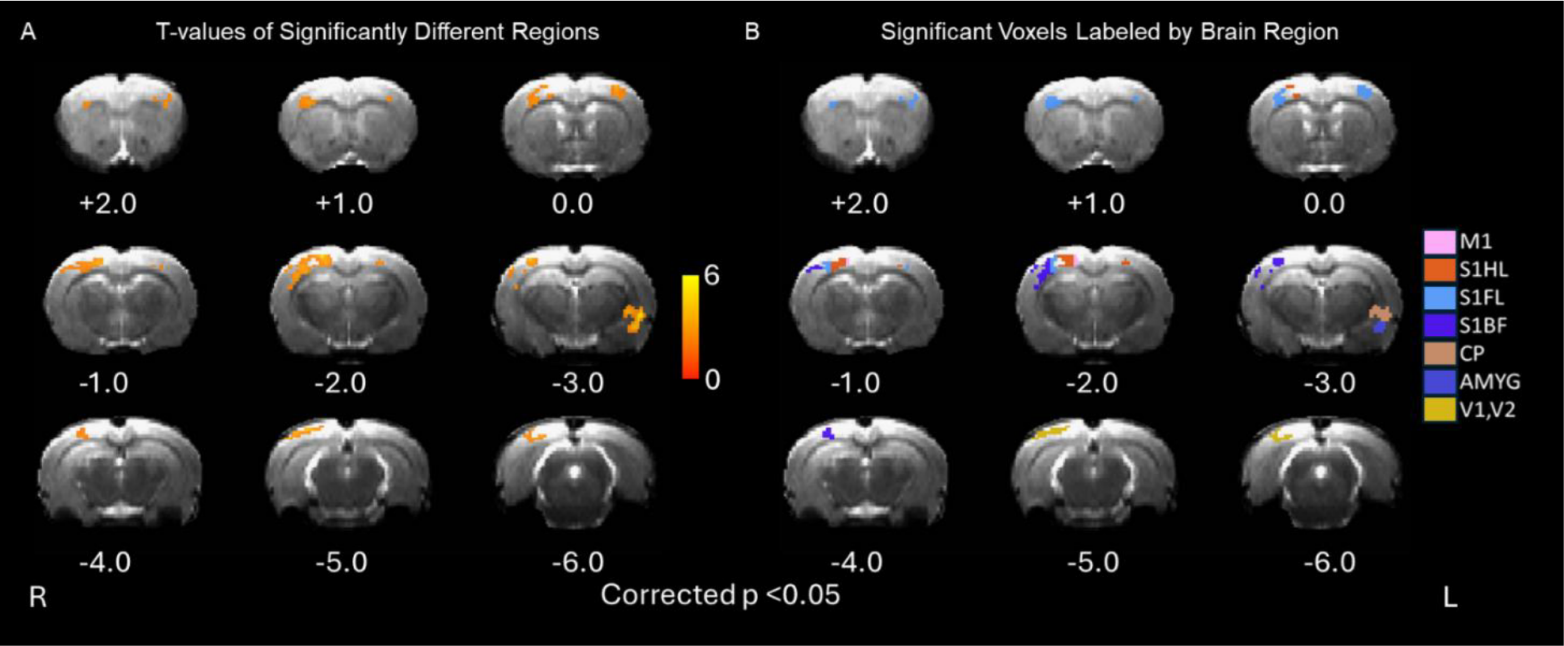
(A) T-statistical maps showing significant alterations in CBV values between the active and sham TMS groups after 5 days of daily hdTBS single-session stimulation (active: N = 8, sham: N = 9, p < 0.05, after multi-comparison correction); significant regions were identified based on rat brain atlas, and are shown in (B).

**Fig. 7.**
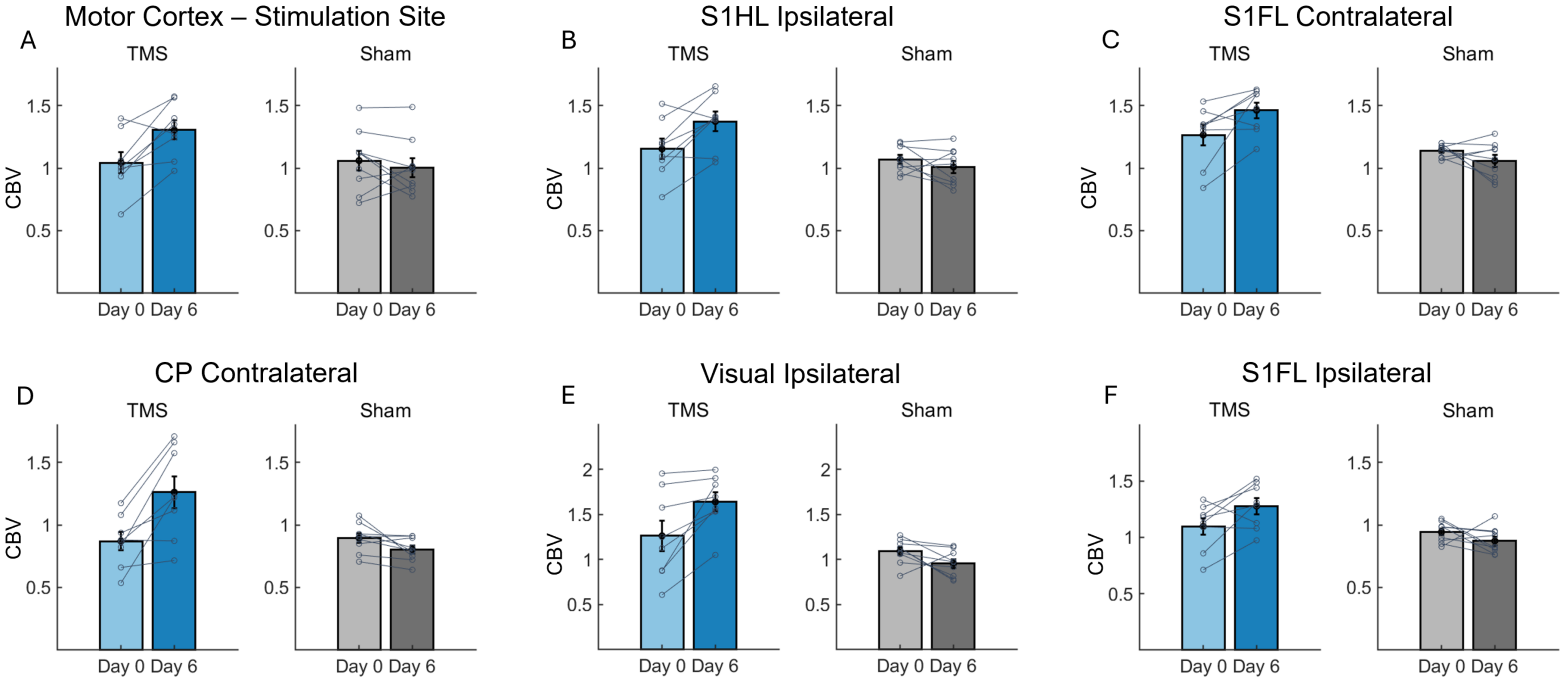
Bar plots showing CBV changes in individual animals at baseline (Day 0) and one day after high-density theta burst stimulation (hdTBS) modulation (Day 6), across the following regions: (A) Motor cortex (M1, the stimulation target), (B) Ipsilateral somatosensory cortex of the hindlimb (S1HL), (C) Contralateral somatosensory cortex of the forelimb (S1FL), (D) Contralateral caudate putamen (CP), (E) Ipsilateral visual cortices (V1/V2), and (F) Ipsilateral S1FL.

Figure 6A show the statistical map comparing ΔCBV resulting from active and sham hdTBS. Areas showing significant differences between these two groups were identified based on the digital rat brain atlas (Paxinos & Watson, 2007), and are color-coded in Figure 6B. Significant differences were observed in the motor cortex (M1, the target site), somatosensory cortex of the hindlimb (S1HL), somatosensory cortex of the forelimb (S1FL), somatosensory barrel cortex (S1BF), caudate putamen (CP), amygdala, and visual cortex (V1, V2). Regional CBV values within each ROI shown in Figure 6B were calculated for each animal. Figure 7 plots ΔCBV on Day 0 and Day 6 in both the TMS group and sham group.

## 4 Discussion

In the present study, leveraging recent technological advancements including the focal TMS coil and hdTBS stimulator, combined with an awake rat model that allows for longitudinal MEP recording, we assessed neural excitability of the rat motor cortex following hdTBS once daily for 5 days. The results reveal that this longitudinal TMS protocol significantly shifted the I-O curve upward, suggesting increased cortical excitability. Additionally, fMRI data demonstrated significant increases in CBV across several regions, including M1, S1HL, S1FL, S1BF, CP, and amygdala, indicating enhanced basal metabolism in these areas due to the longitudinal TMS.

The largest cluster exhibiting a significant increase in basal CBV was located ipsilateral to the stimulation site, centered at bregma -2 mm, corresponding to cortical representation of hindlimb motor cortex and adjacent sensory cortex (Seong et al., 2014). This observation reaffirms the precise targeting ability of the coil. The small cluster in the contralateral sensorimotor region exhibited an increase in CBV, likely influenced by interhemispheric callosal connections originating from the activated ipsilateral hemisphere. Notably, significant CBV increases were also detected in caudate putamen, and amygdala, likely through direct corticocortical and subcortical pathways, because sensorimotor cortex has strong direct projections to caudate putamen (Voorn et al., 2004); studies in rats (Kita & Kitai, 1990), cats (Llamas et al., 1985), monkeys (Amaral & Price, 1984), and humans (Grèzes et al., 2014) have documented direct projections between amygdala and motor cortex.

The imaging results aligned with previous acute stimulation effects in human and support the notion that TMS activation propagates across interconnected network beyond the stimulation loci (Bestmann et al., 2003; Hampson & Hoffman, 2010; Rossini et al., 2015). Collectively, these results strongly suggest that a daily single session of hdTBS for 5 days produces sustained changes in cortical excitability in naïve healthy rats, affecting both the stimulation sites and the connected brain network.

## 5 Comparison with Previous Studies

Studying the effects of TMS was historically initiated in humans. Given the potentials of preclinical models in mechanistic studies, there have been efforts to investigates the neurobiological mechanisms of TMS action employing animal models. While these animal studies have provided important insights into TMS actions, they have been limited by one or more technical constraints. These include focusing solely on acute effects, using large TMS coils that could result in significant off-target effects, or employing electric fields that were insufficient to induce suprathreshold stimulation as observed in humans, or with animals under anesthesia.

In contrast, our current study employed a focal TMS coil specifically designed for rat brains, achieving a focality of 2 mm (Cermak et al., 2020; Meng et al., 2022). Moreover, we administered TMS while the animals were awake, avoiding confounding factors associated with anesthesia—this is especially critical when repeated sessions of TMS have to be administered to produce therapeutic effect, because daily anesthesia of an animal could produce profound effects on the brain, which could mask or interact with the effects produced by TMS. For example, ketamine, a widely used anesthetics, is found to be a potent antidepressant (Berman et al., 2000). We also investigated the effects of longitudinal TMS using established methodologies such as MEP readout, I-O curve assessment, and fMRI mapping—techniques that are commonly used in human research. Additionally, the sensorimotor cortex exhibits a high degree of conservation between rodents and humans, unlike higher-order cortical regions. Together, the methods and findings presented in this article support our rat model as a new platform for further investigating the neurobiological effects of TMS.

## 6 Variability in TMS Response

We observed variability in the responses of animals to daily sessions of hdTBS as shown in Figure 4A and Figure S1. Such variability is not unexpected. High variability in responses to neuromodulation, such as TMS, is commonly seen in both healthy individuals and patients undergoing TMS treatment (Boucher et al., 2021; Downar et al., 2016; McCalley et al., 2021; Ozdemir et al., 2021; Rocchi et al., 2018; Wendt et al., 2023). For instance, a recent study compared MEP responses to iTBS using monophasic versus biphasic pulses. While monophasic iTBS resulted in stronger acute aftereffects than biphasic iTBS, and iTBS on a control site (vertex) had no effect, there was considerable variability among the 30 healthy participants (Fig. 3 of Wendt et al. (2023)). Specifically, four participants exhibited the highest MEP responses in the control condition, while six showed MEP responses that were indistinguishable across the monophasic, biphasic, and control conditions (Fig. S2 in Wendt et al. (2023)). High variability was observed in another study comparing intermittent, continuous, and prolonged TBS protocols (McCalley et al., 2021). The percentage of “responders” in healthy adult rats was comparable to or even higher than that observed in humans.

## 7 Technical Considerations and Limitations

Anesthesia can introduce confounding variables in TMS studies, which can be particularly problematic in longitudinal applications where repeated anesthesia may overshadow or obscure the effects of repeated TMS. For instance, Ketamine, a commonly used anesthetic, is recognized for its potent antidepressant effects and is FDA-approved for treating major depression (Yavi et al., 2022). Gersner and colleagues compared TMS-induced neuroplasticity in awake rats versus rats under isoflurane anesthesia, revealing contrasting effects on neuroplasticity markers such as brain-derived neurotrophic factor (BDNF) levels and GluR1 subunit expression of AMPA receptors (Gersner et al., 2011). In the present study, TMS was administered exclusively in awake rats, avoiding the above confound, but our MRI imaging studies were performed with animals under the anesthesia. Anesthetics, depending on their molecular targets, could influence brain metabolism, affect neurovascular coupling pathways, and alter the arousal state (Brown et al., 2011). The anesthesia protocol, comprising a low dose of dexmedetomidine and isoflurane, has been shown to preserve the synchrony of large-scale brain networks (Brynildsen et al., 2016; Lu et al., 2012), and has become the dominant method in the MRI field (Grandjean et al., 2023; Kint et al., 2020; Ortiz-Rios et al., 2024). Since CBV mapping was conducted with animals under identical anesthesia conditions on Day 0 and Day 6, and both the active and sham hdTBS groups underwent the same anesthesia during imaging, it is not unreasonable to assume that the confounding effects from anesthesia are cancelled out in our statistical analysis.

In our study, MT was only assessed on Day 0, and the same power level was applied during the daily hdTBS sessions. We had previously shown that, in the absence of TMS modulation, MT was relatively stable across days (Cermak et al., 2020). Nevertheless, it is possible that MT could have changed over the course of these hdTBS sessions. Given that TMS pulse power levels are traditionally calibrated based on the MTs, it remains unclear how the observed increase in cortical excitability might be influenced by potential fluctuations in MT.

As our customized stainless-steel electrodes were incompatible with the MRI environment, we were compelled to conduct the CBV MRI experiment and MEP recordings on separate animal groups, limiting our ability to directly correlate changes in basal metabolism with MEP responses within the same individuals, and thereby restricting the investigation of individual variability in TMS responsiveness. Additionally, investigating the long-term durability of the observed brain activity changes would be valuable. In this study, post-hdTBS CBV was mapped at a single time point (Day 6). Future studies should incorporate multiple time points for CBV mapping to better characterize the temporal dynamics of neuroplasticity induced by hdTBS.

In summary, in the current study, we leverage recent technical advances in the lab, including the focal TMS coil, the hdTBS method, and the awake rat TMS model, and investigate the effects of longitudinal TMS on cortical excitability through I-O curve mapping and on systems-level brain activity using CBV mapping. This work establishes a novel platform for further exploration of the neurobiological mechanisms underlying TMS actions.

## Supplementary Material

Supplementary Material

## Data Availability

Raw data and code used in the present study can be provided upon request.
