# Supplementary material for: Functional MRI reveals regional changes of brain activity in rats following longitudinal focal high-density theta burst stimulation (hdTBS)
Source: Imaging Neurosci (Camb). 2025 Jul 25;3:IMAG.a.92. doi: 10.1162/IMAG.a.92 (PMC12330834; doi:10.1162/IMAG.a.92)
Supplement: Supplementary Material [file IMAG.a.92_supp.pdf]

Supplementary Materials

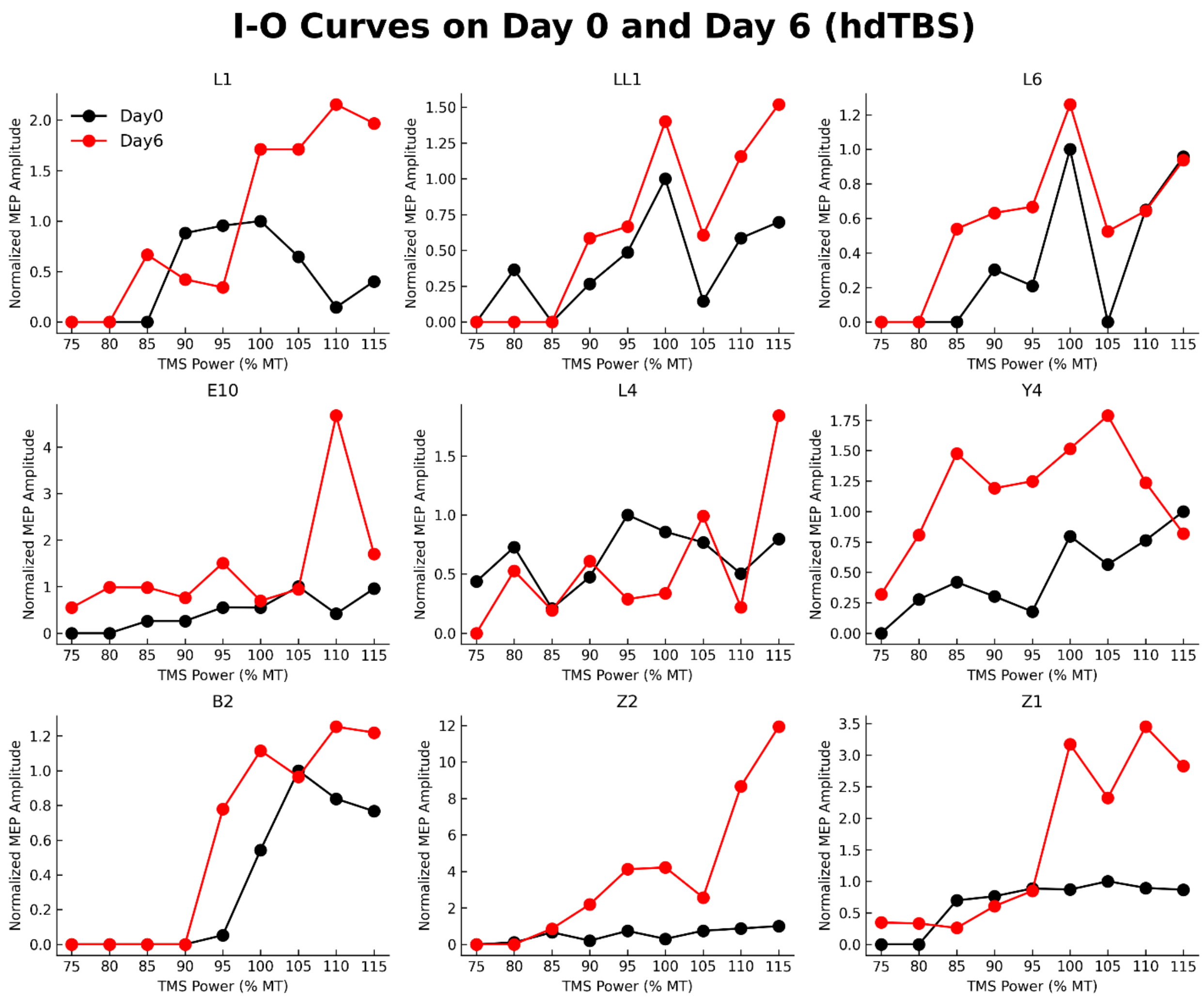

Figure S1. Individual I-O curves on Day 0 and Day 6 for the hdTBS group.

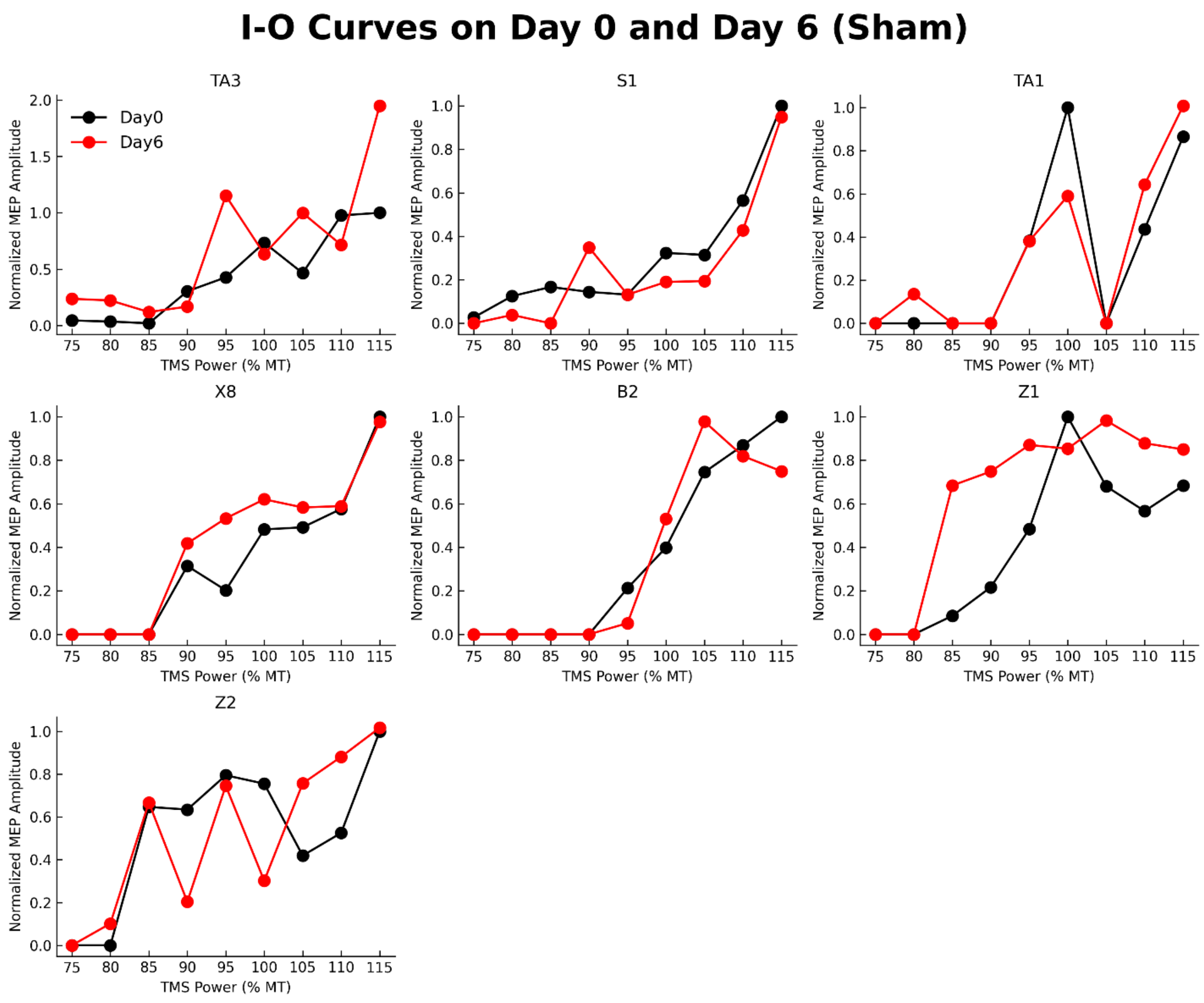

Figure S2. Individual I-O curves on Day 0 and Day 6 for the sham group.
